# Therapeutic Role of Concentrated Growth Factors in the Management of Chronic Heel Tendinopathies: A Prospective Interventional Study

**DOI:** 10.7759/cureus.103880

**Published:** 2026-02-18

**Authors:** Bhanu P Singh, Aman Thakur, Yogesh Singh Parihar

**Affiliations:** 1 Orthopaedics, Gajra Raja Medical College, Gwalior, IND; 2 Orthopaedics, Hindu Rao Hospital, Delhi, IND; 3 Orthopaedics and Traumatology, Gajra Raja Medical College, Gwalior, IND

**Keywords:** achilles tendinopathy, concentrated growth factors, heel tendinopathy, plantar fasciitis, regenerative therapy

## Abstract

Background

Chronic heel tendinopathies, including plantar fasciitis, Achilles tendinopathy, retrocalcaneal bursitis, and Haglund deformity, are among the most common causes of persistent heel pain and functional limitation in adults. Increasing evidence suggests that these conditions represent degenerative failed-healing responses rather than purely inflammatory processes, which explains the limited long-term effectiveness of conventional anti-inflammatory therapies. Concentrated growth factors (CGFs), a second-generation autologous platelet concentrate, provide a dense fibrin matrix enriched with biologically active growth factors that may enhance tendon regeneration and functional recovery.

Objective

To evaluate the clinical efficacy and safety of autologous concentrated growth factor injections in the management of chronic heel tendinopathies refractory to conservative treatment.

Methods

A prospective interventional study was conducted on 50 adult patients (>30 years) diagnosed with chronic heel tendinopathies of more than three months’ duration who had failed standard conservative management. Autologous CGF was prepared from venous blood without anticoagulants and injected locally at the site of maximal tenderness under aseptic conditions. Clinical outcomes were assessed using the Visual Analogue Scale (VAS) for pain and the American Orthopaedic Foot and Ankle Society (AOFAS) score for functional evaluation at baseline, one month, three months, and six months post-injection. Statistical analysis was performed using paired t-tests or Wilcoxon signed-rank tests as appropriate, with a p-value <0.05 considered statistically significant.

Results

The mean baseline VAS score of 7.30 ± 0.89 demonstrated a statistically significant reduction to 5.52 ± 0.65 at one month and to 3.94 ± 0.79 at three months (p < 0.001), with sustained improvement observed at six months. Functional outcomes improved in parallel, with AOFAS scores progressing from poor baseline levels (≤53) to good and excellent categories at subsequent follow-ups. The most pronounced clinical improvement was observed in patients with plantar fasciitis, though favorable outcomes were noted across all diagnostic subgroups. No major complications, including infection or tendon rupture, were recorded.

Conclusion

Autologous concentrated growth factor injections provide significant and sustained pain relief and functional improvement in patients with chronic heel tendinopathies. By addressing the underlying degenerative pathology, CGF therapy represents a safe and biologically rational regenerative treatment option. Further randomized controlled trials with larger sample sizes are recommended to establish definitive treatment guidelines.

## Introduction

Heel pain is among the most frequent musculoskeletal complaints encountered in orthopaedic practice, accounting for approximately 15-20% of foot-related outpatient visits [1]. Chronic heel tendinopathies-including plantar fasciitis, Achilles tendinopathy, retrocalcaneal bursitis, and Haglund deformity-are particularly challenging due to their prolonged clinical course and resistance to conventional therapies [2].

Historically, these conditions were described as inflammatory (“tendinitis”); however, increasing histopathological evidence demonstrates that chronic tendinopathy is predominantly a degenerative failed-healing response, characterized by collagen disorganization, increased proteoglycan content, neovascularization, and tenocyte apoptosis, with minimal inflammatory cell infiltration [3,4]. This paradigm shift explains the limited long-term efficacy of non-steroidal anti-inflammatory drugs and corticosteroid injections, which may provide transient symptom relief but do not address the underlying pathology and may predispose to tendon rupture [5].

Plantar fasciitis alone affects up to 10% of the population during their lifetime and is a leading cause of inferior heel pain in both athletic and sedentary individuals [6]. Similarly, Achilles tendinopathy is prevalent in middle-aged individuals and athletes, frequently leading to functional limitation and reduced quality of life [7].

Regenerative medicine has gained prominence as a therapeutic strategy aimed at restoring tendon structure and function. Platelet-derived biologics deliver autologous growth factors that modulate angiogenesis, fibroblast proliferation, collagen synthesis, and extracellular matrix remodeling [8]. Concentrated growth factors (CGFs) represent an advanced platelet concentrate produced without anticoagulants, yielding a dense fibrin matrix with sustained release of biologically active mediators such as PDGF, TGF-β, VEGF, and IGF [9].

Compared with platelet-rich plasma (PRP), CGFs provide a higher fibrin density and prolonged growth factor availability, potentially enhancing tissue regeneration [10]. Given the degenerative nature of heel tendinopathies, CGFs may offer a biologically rational treatment option. This study evaluates the clinical effectiveness of autologous CGF injections in chronic heel tendinopathies.

## Materials and methods

Study design and setting

The study was conducted over a 6-month period from July 2022 to December 2022 in the Department of Orthopaedics at a tertiary care government hospital. Ethical approval was obtained from the Institutional Ethics Committee prior to patient recruitment (IEC approval date: 16 June 2022). Written informed consent was obtained from all participants. All procedures were performed in accordance with the ethical standards of the Declaration of Helsinki.

Study population

Patients presenting to the orthopaedic outpatient department with complaints of chronic heel pain were screened for eligibility. A total of 50 patients who fulfilled the inclusion and exclusion criteria were consecutively enrolled using purposive sampling. The sample size for the present study was calculated based on the expected improvement in pain scores following biological injection therapy in chronic heel tendinopathies. Previous literature evaluating platelet-based regenerative injections in plantar fasciitis has demonstrated a mean reduction in visual analogue scale (VAS) score of approximately 2.7 with a standard deviation (SD) of 0.5 at short-term follow-up.

The minimum required sample size was calculated to detect a statistically significant difference in mean VAS scores with: Power of study (1 − β): 80%, Level of significance (α): 5%, two-tailed test.

The standard formula used for sample size calculation for comparison of means was:

The sample size for the present study was calculated based on the expected improvement in pain scores following biological injection therapy in chronic heel tendinopathies. Previous literature evaluating platelet-based regenerative injections in plantar fasciitis has demonstrated a mean reduction in Visual Analogue Scale (VAS) score of approximately 2.7 with a standard deviation (SD) of 0.5 at short-term follow-up.

The minimum required sample size was calculated to detect a statistically significant difference in mean VAS scores with:

Power of study (1 − β): 80%; Level of significance (α): 5%

Two-tailed test

The standard formula used for sample size calculation for comparison of means was:

$n = \frac{Z_{1-\alpha/2}^2 \times \sigma^2}{d^2} ​$

Where:

n = required sample size; Z _1−α/2_ = standard normal deviate corresponding to 95% confidence interval (1.96); σ = standard deviation of the outcome variable (0.5); d = allowable error (precision), taken as 15% of the mean improvement

On substitution of values: n= 42. Thus, the minimum required sample size was approximately 42 patients. To account for an anticipated 20% loss to follow-up, the adjusted sample size was calculated as: n_adjusted_=42/ 0.8=52.5. Accordingly, a final sample size of 50 patients was enrolled in the study to ensure adequate statistical power while maintaining feasibility.

Study objectives

The objectives of the present study were as follows:

Primary Objective

To evaluate the effectiveness of autologous concentrated growth factor (CGF) injections in reducing pain severity in patients with chronic heel tendinopathies, as measured by the change in Visual Analogue Scale (VAS) score from baseline to 3 and 6 months of follow-up.

Secondary Objectives

To assess functional improvement following CGF injection using the American Orthopaedic Foot and Ankle Society (AOFAS) Ankle-Hindfoot Score at 1, 3, and 6 months.

To evaluate the safety profile of autologous CGF injections by documenting procedure-related complications during the follow-up period.

Inclusion criteria

Patients were included in the study if they met all of the following criteria: Age greater than 30 years, irrespective of sex, clinically diagnosed cases of chronic heel tendinopathies, including plantar fasciitis, achilles tendinopathy, retrocalcaneal bursitis, or Haglund deformity, duration of symptoms exceeding three months, failure of prior conservative management, including rest, analgesics, physiotherapy, footwear modification, or orthotics, baseline pain severity ≥ 5 on a 10-point Visual Analogue Scale (VAS), baseline AOFAS score ≤ 53, indicating poor functional status.

Exclusion criteria

Patients were excluded if any of the following were present: Local infection or skin lesion at the injection site, previous trauma or surgical intervention involving the affected heel, systemic inflammatory or autoimmune diseases (e.g., rheumatoid arthritis, seronegative spondyloarthropathies), known platelet dysfunction, coagulopathy, or bleeding disorders, recent intake of antiplatelet drugs, aspirin, or NSAIDs within 72 hours, or corticosteroids within four weeks prior to injection, patients unwilling to participate or unable to comply with follow-up protocol.

Baseline clinical evaluation

At presentation, detailed demographic and clinical data were recorded, including age, sex, occupation, side involved, duration of symptoms, and prior treatment history. A thorough clinical examination was performed to confirm the diagnosis and to exclude other causes of heel pain. Imaging studies, such as plain radiographs or ultrasonography, were used selectively to rule out alternate diagnoses where clinically indicated. Pain severity was assessed using the Visual Analogue Scale (VAS), and functional status was evaluated using the American Orthopaedic Foot and Ankle Society (AOFAS) Ankle-Hindfoot Score, which includes pain, function, and alignment components.

Preparation of concentrated growth factors (CGF)

Autologous concentrated growth factors were prepared using a standardized protocol. Under aseptic precautions, 8 mL of venous blood was drawn from the antecubital vein and collected in a sterile glass tube without an anticoagulant. The blood sample was gently inverted 8-10 times and allowed to rest in an upright position for approximately 30 minutes. 

Autologous concentrated growth factors were prepared using a dedicated centrifugation system with a fixed-angle rotor of radius 110 mm. Venous blood (8 mL) was centrifuged at 3400 rpm, corresponding to an approximate relative centrifugal force (RCF) of 708 × g, calculated using the standard formula:

$\mathrm{RCF} = 1.118 \times 10^{-5} \times r \times (\mathrm{rpm})^2$

Where 'r' represents the rotor radius in centimeters.

The sample was then centrifuged using a dedicated CGF centrifugation device at 3400 revolutions per minute (rpm) for 10 minutes. This process resulted in separation into three distinct layers: Upper layer: Concentrated growth factor-rich plasma. Middle layer: Dense fibrin-rich thixotropic gel. Lower layer: Red blood cell fraction. The upper CGF fraction, rich in platelet-derived growth factors, was carefully aspirated using a sterile syringe and prepared for injection. 

Volume of CGF Injected: A standardized volume of 2.5-3.0 mL of CGF was injected per patient, irrespective of diagnostic subgroup, ensuring consistency across all cases.

Injection technique

The injection procedure was performed in a minor procedure room under strict aseptic conditions. The site of maximal tenderness was identified clinically and marked. After skin preparation and draping, the prepared CGF was injected directly into the pathological site using a sterile technique. No local anesthetic was mixed with the CGF to avoid potential interference with platelet activation. Following the injection, a sterile dressing was applied, and a light compression bandage was used. Patients were observed for a short period post-procedure to monitor for immediate adverse reactions.

Post-procedural care and rehabilitation

Patients were advised to rest for 48 hours following the injection. Analgesia was provided using paracetamol, and NSAIDs were avoided to prevent inhibition of platelet-mediated healing. Patients were instructed in a home-based stretching and strengthening program, focusing on the gastrocnemius-soleus complex and plantar fascia, which was initiated after the acute post-injection period.

A standardized rehabilitation protocol was followed for all patients:

Days 1-3: Relative rest and activity modification

Week 1-2: Gentle stretching exercises of the gastrocnemius-soleus complex (3 sets of 10 repetitions, twice daily)

Week 3-6: Progressive eccentric strengthening exercises (3 sets of 15 repetitions, once daily)

After 6 weeks: Gradual return to normal activity as tolerated

NSAIDs were avoided throughout the rehabilitation period to prevent interference with platelet-mediated healing.

Post-procedural analgesia was provided using paracetamol alone, and non-steroidal anti-inflammatory drugs (NSAIDs) were strictly avoided to prevent interference with platelet-mediated and growth factor-driven healing processes.

Follow-up and outcome assessment

Patients were followed up at one month, three months, and six months post-injection. At each follow-up visit, pain was reassessed using the VAS, functional outcome was evaluated using the AOFAS score, and patients were examined for any local or systemic complications.

Statistical analysis

Data were entered into Microsoft Excel and analyzed using Statistical Package for Social Sciences (SPSS) version 21.0. Continuous variables were expressed as mean ± standard deviation (SD), and categorical variables were expressed as frequency and percentage. Normality of data distribution was assessed using the Kolmogorov-Smirnov test. Paired t-test was used for normally distributed data, and the Wilcoxon signed-rank test was used for non-parametric data to compare outcomes across follow-up intervals. A p-value < 0.05 was considered statistically significant.

## Results

Baseline demographic and clinical characteristics

A total of 50 patients with chronic heel tendinopathies were included in the analysis. The mean age of the study population was 45.46 ± 7.8 years (range: 31-60 years). There was a male predominance (31 males, 62%) compared to females (19, 38%). The most common diagnosis was plantar fasciitis, accounting for 54% of cases, followed by Haglund deformity and retrocalcaneal bursitis (16% each), and Achilles tendinopathy (14%). The right side was more frequently involved (58%) than the left (42%).

Pain assessment (VAS score)

At baseline, patients reported moderate to severe pain, with a mean VAS score of 7.30 ± 0.89. Following autologous CGF injection, there was a progressive and statistically significant reduction in pain intensity at each follow-up interval (Table 1). At one month, the mean VAS score decreased to 5.52 ± 0.65, representing a mean reduction of 1.78 points from baseline (p < 0.001). This improvement further increased at three months, with the mean VAS score reducing to 3.94 ± 0.79, corresponding to a total mean reduction of 3.36 points from baseline (p < 0.001). The pain relief achieved at three months was sustained at six months, indicating durable clinical benefit. These findings demonstrate a clinically meaningful and statistically significant analgesic effect of CGF injections in chronic heel tendinopathies. The reduction in VAS scores across follow-up intervals was statistically significant on paired t-test analysis (t = 12.46 at one month and t = 18.21 at three months; p < 0.001).

**Table 1:** Comparative analysis of VAS scores over follow-up Data are presented as Mean ± Standard Deviation (SD). Statistical analysis was performed using a paired t-test. A p-value < 0.05 was considered statistically significant.

Time Point	Mean Change from Baseline	Mean VAS ± SD	t-value	p-value
Baseline	—	7.30 ± 0.89	—	—
1 month	−1.78	5.52 ± 0.65	12.46	<0.001
3 months	−3.36	3.94 ± 0.79	18.21	<0.001

Functional outcome (AOFAS score)

Functional assessment using the AOFAS score showed significant improvement following CGF therapy (Table 2). At baseline, most patients had poor functional scores (≤53), reflecting substantial pain-related disability and limitation in daily activities. At one month, there was a noticeable improvement in AOFAS scores, indicating early functional recovery. By three months, the majority of patients transitioned into the good functional category, demonstrating improved walking ability, reduced pain during activity, and enhanced ankle-foot function. These gains were maintained at six months, suggesting sustained functional restoration. Overall, CGF injections resulted in progressive functional improvement, paralleling the reduction in pain scores. A p-value < 0.05 was considered statistically significant.

**Table 2:** Functional outcome based on AOFAS score AOFAS: American Orthopaedic Foot and Ankle Society

Time Point	Mean AOFAS Score ± SD	Functional Interpretation
Baseline	49.2 ± 3.1	Poor
1 month	63.4 ± 4.6	Fair
3 months	78.6 ± 5.2	Good
6 months	82.9 ± 4.8	Good–Excellent

Diagnosis-wise response to CGF therapy

A subgroup trend analysis revealed consistent improvement across all diagnostic categories (Table 3). Patients with plantar fasciitis demonstrated the greatest reduction in pain and functional improvement, followed by those with Achilles tendinopathy. Patients with retrocalcaneal bursitis and Haglund deformity also showed moderate but clinically meaningful improvement. Although the study was not powered for formal intergroup statistical comparison, the uniform positive trend across diagnoses supports the broad applicability of CGF therapy in heel tendinopathies.

**Table 3:** Diagnosis-wise clinical response to CGF injection. CGF: Concentrated growth factors

Diagnosis	Mean VAS Reduction ± SD	Mean AOFAS Improvement ± SD
Plantar fasciitis	3.8 ± 0.6	32.4 ± 4.9
Achilles tendinopathy	3.1 ± 0.5	26.7 ± 4.3
Retrocalcaneal bursitis	2.9 ± 0.4	24.5 ± 3.8
Haglund deformity	2.7 ± 0.4	23.8 ± 3.6

Safety and complications

No major adverse events, including infection, neurovascular injury, allergic reaction, or tendon rupture, were observed during the follow-up period. All patients tolerated the procedure well, confirming the favorable safety profile of autologous CGF injections.

## Discussion

Chronic heel tendinopathies constitute a significant cause of persistent pain and functional impairment in adults, often leading to reduced mobility, decreased occupational productivity, and diminished quality of life. These disorders frequently involve the plantar fascia and Achilles tendon, structures that are subjected to repetitive mechanical loading and age-related degenerative changes [1]. The high prevalence and chronicity of these conditions, particularly in middle-aged individuals, underscore the need for effective and durable treatment strategies [2].

Historically, heel tendinopathies were considered inflammatory in origin; however, increasing histopathological evidence has demonstrated that chronic tendinopathy represents a degenerative, failed-healing response, rather than an ongoing inflammatory process [3]. Structural changes such as collagen fiber disorganization, increased proteoglycan content, neovascularization, and tenocyte apoptosis have been consistently observed, with minimal inflammatory cell infiltration [4]. This paradigm shift has important therapeutic implications, as it explains the limited long-term efficacy of anti-inflammatory agents and corticosteroid injections, which primarily target inflammation rather than tissue regeneration [5].

Plantar fasciitis, the most common diagnosis in the present study, affects up to 10% of the population at some point during their lifetime and accounts for a substantial proportion of heel pain presentations [6]. Similarly, Achilles tendinopathy is a prevalent condition, particularly in physically active and middle-aged populations, and is associated with prolonged disability and a high recurrence rate [7]. Conventional conservative measures, although effective in many patients, fail to provide sustained relief in a subset of cases, necessitating exploration of regenerative treatment options.

The rationale for the use of platelet-derived biologics in tendinopathy is based on their ability to deliver high concentrations of growth factors that regulate angiogenesis, fibroblast proliferation, collagen synthesis, and extracellular matrix remodeling- key processes in tendon healing [8]. Concentrated growth factors represent an advanced form of autologous platelet concentrate, characterized by a dense fibrin matrix and sustained release of biologically active mediators such as platelet-derived growth factor (PDGF), transforming growth factor-β (TGF-β), vascular endothelial growth factor (VEGF), and insulin-like growth factor (IGF) [9].

In the present study, autologous CGF injections resulted in statistically and clinically significant reductions in pain, as evidenced by progressive improvement in VAS scores from baseline through all follow-up intervals. The magnitude of pain reduction exceeded the minimum clinically important difference commonly reported for chronic foot and ankle conditions, indicating meaningful symptom relief. Functional outcomes assessed using the AOFAS score improved in parallel, reflecting enhanced mobility, reduced activity-related pain, and improved overall foot and ankle function. 

The sustained improvement observed at six months suggests that CGF therapy may exert a durable regenerative effect, rather than a transient analgesic response. This finding is particularly relevant when contrasted with prior studies evaluating platelet-rich plasma (PRP) and autologous blood injections, which have reported variable and often inconsistent outcomes [10]. Systematic reviews have highlighted methodological heterogeneity, lack of standardization in PRP preparation, and inconsistent growth factor concentrations as major limitations contributing to mixed clinical results [11]. While significant improvements in pain and functional scores were observed following CGF injection, the single-arm design of this study limits causal inference. The findings therefore support within-group clinical improvement and short-term safety, rather than definitive efficacy, superiority, or structural tissue regeneration attributable solely to CGF therapy.

Compared with PRP, CGFs provide a more stable fibrin scaffold and a prolonged release of growth factors, which may enhance cellular migration, angiogenesis, and collagen remodeling over an extended period [12]. This biological advantage may explain the favorable outcomes observed in the present study, particularly in patients with plantar fasciitis, who demonstrated the greatest improvement in both pain and function.

Previous clinical studies investigating platelet-based therapies in plantar fasciopathy have yielded conflicting results. While some authors have reported no significant benefit over corticosteroid injections or exercise therapy, others have demonstrated meaningful long-term improvements in pain and functional scores [13]. The variability in outcomes underscores the importance of biologic preparation methods and supports the potential superiority of CGFs as a more consistent regenerative modality.

Experimental and clinical data have demonstrated that CGFs release higher concentrations of growth factors compared to conventional PRP, with enhanced angiogenic and tissue-regenerative potential [14]. These properties are particularly advantageous in chronic degenerative conditions, where intrinsic healing capacity is compromised. Furthermore, unlike corticosteroid injections, which have been associated with plantar fascia rupture and tendon weakening, CGF therapy demonstrated a favorable safety profile, with no major complications observed in the present cohort [15].

Study limitations

Despite the encouraging clinical outcomes observed, several limitations of this study must be acknowledged. First, the absence of a control or placebo group limits the ability to definitively attribute the observed improvements solely to the biological effect of concentrated growth factor (CGF) injections. Placebo response and natural disease progression, particularly in chronic heel tendinopathies, may have contributed to symptom improvement. Additionally, the use of patient-reported outcome measures such as VAS and AOFAS scores introduces the possibility of measurement and observer bias, despite their widespread validation and clinical relevance.

Second, although a standardized protocol for CGF preparation and administration was followed, biologic variability inherent to autologous blood-derived products cannot be completely eliminated. Differences in individual platelet concentration, growth factor release kinetics, and fibrin matrix composition may affect reproducibility across centers and patient populations. This limitation is intrinsic to regenerative therapies and should be considered when interpreting and generalizing the results.

Third, the study included a heterogeneous group of heel tendinopathies, including plantar fasciitis, Achilles tendinopathy, retrocalcaneal bursitis, and Haglund deformity. While all these conditions share a degenerative pathophysiological basis, differences in tissue involvement and biomechanical loading patterns may influence treatment response. The sample size was not powered to permit robust subgroup analysis, which limits condition-specific conclusions.

Finally, the single-center design and relatively modest sample size restrict external validity. Larger, multicenter randomized controlled trials incorporating standardized imaging-based diagnostic criteria, objective outcome measures, and comparator groups are required to confirm these findings and to establish definitive clinical recommendations.

## Conclusions

Chronic heel tendinopathies are degenerative musculoskeletal disorders that commonly lead to persistent pain, functional limitation, and reduced quality of life. Conventional conservative treatments, largely aimed at symptom control, often fail to provide sustained relief in a subset of patients because they do not adequately address the underlying degenerative pathology. In this prospective interventional study, autologous concentrated growth factor (CGF) injections resulted in statistically significant and clinically meaningful improvements in both pain and functional outcomes in patients with chronic heel tendinopathies refractory to standard conservative management. The progressive reduction in pain scores and parallel improvement in functional assessment across all follow-up intervals suggest that CGF therapy produces a sustained therapeutic effect rather than a transient analgesic response.

Autologous concentrated growth factor injections were associated with significant improvements in pain and function and demonstrated a favorable safety profile in patients with chronic heel tendinopathies. However, given the observational single-arm design, these findings should be interpreted as supportive evidence of potential clinical benefit, and not as proof of efficacy or regenerative superiority. Controlled randomized studies are required to establish causality and comparative effectiveness. CGF injections demonstrated a favorable safety profile, with no major complications observed during follow-up, positioning CGF therapy as a promising regenerative alternative to corticosteroid injections, which are associated with short-term benefit and potential structural weakening of tendon tissue. Although the results are encouraging, larger randomized controlled trials with standardized imaging-based outcome measures and longer follow-up durations are required to establish definitive clinical guidelines. Overall, autologous CGF injection represents a safe, biologically rational, and effective regenerative treatment option for chronic heel tendinopathies, with the potential to reduce the need for surgical intervention and improve patient-centered outcomes.
